# Evaluating the Effect of Acetazolamide on the Prevention of Post-operative Acute Kidney Injury after Coronary Artery Bypass Grafting Surgery: A Randomized, Open-labeled Clinical Trial

**DOI:** 10.22037/ijpr.2021.115334.15323

**Published:** 2021

**Authors:** Golnaz Afzal, Zahra Ansari Aval, Mahmoud Beheshti Monfared, Hamed Khesali, Shadi Ziaie, Saghar Barati, Farzaneh Dastan

**Affiliations:** a *Department of Clinical Pharmacy, School of Pharmacy, Shahid Beheshti University of Medical Sciences, Tehran, Iran. *; b *Clinical Research and Development Center at Shahid Modarress Hospital, Department of Cardiac Surgery, Shahid Beheshti of Medical Sciences, Tehran, Iran. *; c *Department of Cardiovascular Surgery, Shahid Modarres Hospital, Shahid Beheshti University of Medical Sciences, Tehran, Iran. *; d *Department of Nephrology and Kidney Transplantation, Shahid Labbafinejad Medical Center, Shahid Beheshti University of Medical Sciences, Tehran, Iran. *; e *Chronic Respiratory Diseases Research center, National Research Institute of Tuberculosis and Lung Diseases (NRITLD), Shahid Beheshti University of Medical Sciences, Tehran, Iran.*

**Keywords:** Aortocoronary Bypasses, Carbonic Anhydrase Inhibitor, Diamox, Renal Insufficiency, Acute, Renal Failure, Acute

## Abstract

Acute kidney injury (AKI) is a common complication after coronary artery bypass grafting (CABG) surgery and can be linked to the increased morbidity and mortality. Therefore, in the present study, the effect of preoperative administration of acetazolamide was evaluated to investigate whether it could prevent occurrence of post-operative AKI after CABG surgery. In this randomized controlled clinical trial, 130 patients who were candidates to undergo elective CABG surgery from January 21, 2020 to February 8, 2021 were randomly allocated to intervention group (receiving 500 mg of acetazolamide orally 2 h preoperatively) and control group. The patients were evaluated for AKI based on the kidney disease- improving global outcomes (KDIGO) criteria based on their serum creatinine (SCr) level and urine output until 7 days postoperatively. There was no significant difference in baseline demographics between the two groups. The total incidence of AKI was measured as 43%. Analysis of post-operative AKI incidence showed no statistically significant difference between the two groups (*P* = 0.860). Mean post-operative SCr level on day 1 was significantly higher in the acetazolamide group (*P *= 0.036). A significant difference was found in length of hospitalization stay between the groups, which was higher in the control group (*P* = 0.006). Our results did not demonstrate a significant protective effect of acetazolamide on incidence of post-operative AKI in the patients undergone elective on-pump CABG surgery.

## Introduction

Coronary artery bypass grafting (CABG) surgery is a treatment of choice for many patients with coronary artery disease (1). Patients undergoing CABG surgery are at risk of serious complications (2) , such as acute kidney injury (AKI), with an incidence rate ranging between 5 - 42% (2, 3). AKI causes an increase in-hospital mortality by 10–30%, which can be increased even by 40–60% if dialysis is required (4). Many factors have been suggested in preventing AKI after cardiac surgery, including minimizing the usage of ischemic and nephrotoxic agents, decreasing renal vascular resistance, and inducing alkalinization of tubular fluid (5, 6).

In this regard, several studies have investigated the effect of pharmacological interventions to prevent AKI, such as N-acetylcysteine, mannitol, vitamin E, steroid, statin, dopamine, fenoldopam, and sodium bicarbonate. However, many of these preventive strategies failed to decrease the incidence rate of AKI (7-12), or the findings of these studies might not be very reliable because of the minor or poor quality of these studies (13, 14). Therefore, there is an increasing interest in decreasing post-operative AKI in order to improve clinical outcomes. There is a large body of growing evidence showing that acetazolamide can play a role in preventing AKI (15-18). Various mechanisms of acetazolamide, including alkalinizing renal tubular ﬂuid (17), scavenging reactive oxygen species (16), and improving renal circulation (18) have been investigated in the previous studies. However, the role of perioperative administration of acetazolamide is unclear in kidney function after surgery. Moreover, the previous studies are heterogeneous regarding doses and acetazolamide administration, length of follow-up, outcome ascertainment methods, and other characteristics in the studied human subjects or animal models that have led to inconsistent results. Therefore, the present study was conducted to evaluate the effects of acetazolamide on AKI prevention after CABG surgery.

## Experimental

This study was approved by the Ethics Committee of the Shahid Beheshti University of Medical Sciences, Tehran, Iran (Ethics code: IR.SBMU.PHARMACY.REC.1399.146) with registry code of IRCT20151227025726N21 in the Iranian registry of clinical trials (IRCT). Informed written consent was obtained from all the patients before enrollment in the study.

The present randomized, open-labeled clinical trial was conducted in the Shahid Modarres Hospital—a tertiary university hospital in Tehran, Iran, on the patients who were candidates to undergo the CABG surgery from January 21, 2020, to February 8, 2021. The sample size was calculated based on 80% of power and 95% of confidence level; and significance level (α) was assumed to be 0.05. The highest number was selected for sample size.

All the patients aged more than 18 years old and admitted to the hospital to undergo elective on-pump CABG surgery were enrolled in the study. 

Exclusion criteria were patients with 1) a history of hypersensitivity to acetazolamide, and any sulfonamide compounds; 2) stages IV and V of chronic kidney disease based on the modified diet in renal disease (MDRD) equation; 3) liver failure (Child-Pugh stages B and C); 4) left ventricular ejection fraction (LVEF) lower than 30 %; 5) hypokalemia (potassium <3.3 mmol/ L); 6) hyponatremia (sodium < 135 mmol/ L); 7) gout attacks; 8) metabolic acidosis; 9) and the need for undergoing emergency CABG surgery. 

An online statistical computing web program (www. Sealedenvelope.com) was utilized to randomize the assignment of the patients in acetazolamide or control groups. The patients in acetazolamide group received 500 mg acetazolamide tablet (Darou Pakhsh, Iran) 2 h before surgery, while the control group did not receive acetazolamide. 

The patients’ demographic data, medical and drug history, as well as laboratory data were also documented. 

The basic kidney function was monitored before and after CABG surgery. Serum creatinine concentration (SCr) was usually measured daily using Jaffe chemistry techniques with a total imprecision of < 6%. Every patient’s creatinine value was analyzed. Both relative and absolute increases in SCr concentration were used to diagnose and classify AKI stages according to the kidney disease-improving global outcomes (KDIGO) criteria; daily urine output and the need for dialysis were also recorded (Table 1).

Induction and maintenance of anesthesia, and surgery were conducted using the same method in both groups. All the patients in both groups received the same standard surgery protocol of intravenous fluid type based on their weight, utilizing ringer, albumin, heparin, corticosteroid, diuretic, and cefazolin as preoperative antibiotic prophylaxis, which is designed for patients underwent on-pump cardiopulmonary bypass surgery according to the the patient’s hemodynamic status. One cardiac surgeon performed all the CABG surgeries according to the standard practice guidelines. Perioperative data, such as anesthesia time, cross-clamping time, cardiopulmonary bypass time, intubation time, need for intravenous fluid (IV), inotropes, blood transfusion, and nephrotoxic drug were also recorded. All the patients were transferred to the intensive care unit (ICU) according to standard protocol. C-reactive protein (CRP) level and LVEF were measured in all the patients at baseline (on morning before surgery) and 24 and 48 h after surgery, respectively. Levels of CRP were measured using the CRP-latex immunoturbidometric assay (CRP-LIA).

Adverse reactions regarding the use of acetazolamide and surgery complications were evaluated and described in each group based on the Naranjo scale.

Patients were followed up for 7 days to evaluate AKI after CABG surgery based on KDIGO criteria as a primary outcome (19). All the patients were followed up until discharge.

Secondary outcomes, duration of post-operative mechanical ventilation, ICU, hospital length of stay need for dialysis, and mortality rate were evaluated.

*Statistical Analysis*

All the statistical analyses were performed using SPSS software for Windows (Version 23.0; SPSS Inc., Chicago, IL, USA). Categorical and nominal variables were expressed as frequency (%) and were compared using the Chi-Square test. Continuous variables were tested for normal distribution by the Kolmogorov–Smirnov test. Data were expressed as means/standard deviations or median, interquartile ranges (25th and 75th percentile), depending on the variable’s parametric or non-parametric distribution. So that, if our data followed a normal distribution, parametric tests were used; otherwise, non-parametric methods were used to compare them. P-values < 0.05 were considered as statistically significant.

## Results

**Figure 1:**
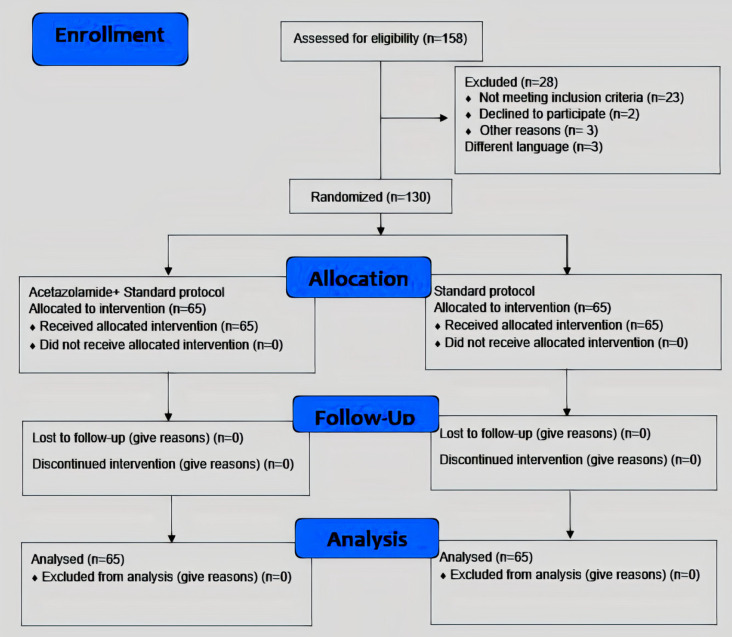
CONSORT Flow Diagram

During the study period, 158 patients were assessed for eligibility; 28 of them were excluded from the study for not meeting inclusion criteria, declining to participate, or having a different language. Among 130 patients randomly assigned in both study groups (acetazolamide group, n = 65; control group, n = 65), all of them underwent all the study procedures (Figure 1). 

No significant differences were found between the control and acetazolamide groups regarding baseline demographic and clinical characteristics, such as age, sex, body mass index (BMI), coexisting medical conditions, preoperative drugs, and LVEF (Table 2).

Table 3 shows biochemical data of the patients in the control and acetazolamide groups before and after CABG surgery. There were no signiﬁcant differences in baseline SCr concentration, blood pH, and serum bicarbonate level between the two groups. Mean ± SD of SCr levels measured 24 h after CABG surgery was significantly lower in the patients who did not receive acetazolamide than those treated with it. Additionally, the mean absolute change of SCr concentration on the first day after CABG surgery was significantly higher in the acetazolamide group. Blood pH and serum bicarbonate levels were significantly lower in the acetazolamide group than the control group on the first day after CABG surgery.

As demonstrated in Table 4, AKI was diagnosed in 57 patients (43%). There was no significant difference in the incidence of AKI between the patients who received acetazolamide (44.61%) *vs*. the control group (43.07%) (*P* = 0.860). Patients who received acetazolamide had a significantly lower median length of hospital stay than those who did not receive it (8 days compared to 9 days, *P* = 0.006).

**Figure 2:**
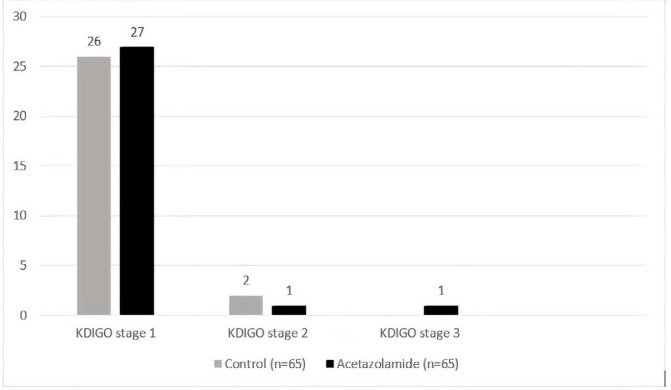
AKI stage based on KDIGO in the acetazolamide and the control group

There was no significant difference in KDIGO AKI staging between the two groups (Figure 2). A total of 53 patients developed stage 1 of AKI. Among 29 patients in the acetazolamide group who developed AKI, SCr concentration was increased by 0.3 mg/dl or more within 48 h in 27 patients (93%). There was one patient in the acetazolamide group with AKI who progressed to stage 3.

Intra- and post-operative characteristics and complications of the patients in both groups are shown in Table 5. There was no difference in the need for inotropes, packed red blood cells, cross-clamping time, and cardiopulmonary bypass time between both groups. 

There was no significant difference in post-operative LVEF, serum CRP level, adverse effects, and surgery complications between the two groups.

## Discussion

In this randomized controlled trial, our results showed that post-operative AKI incidence was not significantly different between acetazolamide and control groups. Also, the need for mechanical ventilation, dialysis, admission to the ICU, or mortality rate did not differ significantly between the two groups. Length of hospital stay was significantly lower in the acetazolamide group.

 The incidence of AKI, which occurred post-CABG surgery, is common and increases the risk of post-operative complications and mortality (20). Despite the advancements achieved in cardiac surgery and post-operative care, the incidence of AKI has increasingly become a great concern for all health professionals (21) as it is well acknowledged that to date, there are no pharmacological interventions proven to reduce the incidence of AKI after CABG surgery (22, 23). Therefore, the present study was conducted to evaluate the acetazolamide’s effects on ongoing kidney injury after CABG surgery.

Based on the results, there was no difference in the incidence of AKI between patients in the acetazolamide and the control group. The previously published studies have demonstrated that acetazolamide administration could be useful in preventing renal injury through different mechanisms, including reducing oxidative state, potential vasodilatory effect on renal circulation, and alkalinization of renal tubular fluid (16, 18 and 24-27). Shamash et al., showed that acetazolamide could play a renoprotective role by inhibiting renal tubular reabsorption of bicarbonate, inducing alkalinization, and decreasing precipitation of acidic drug in renal tubule (17). Additionally, Horita *et al*., (27) and Yu An *et al*., (18) reported that direct infusion of 1 g of acetazolamide into the patient’s main renal arteries and oral administration of 60 mg/kg/day of acetazolamide in mice could increase renal circulations by vasodilatory effect, respectively. This contrast in the results could be related to the difference in the route of administration (local infusion and intravenous (IV)), dosing, course, repeated exposure of acetazolamide, study population, and studying critically or stable ill individuals, which varied widely among these studies (27).

Surprisingly, our results showed that both SCr level and its change compared to baseline were significantly higher in the acetazolamide group than the control group on the first day after CABG surgery.

Several case reports have demonstrated an acute increase in SCr concentration following short courses of acetazolamide administration. All the cases have been presented with azotemia and back pain, as well as progressiveanuria. Those with radiographic evidence of intratubular obstruction revealed cellular debris, mucosal swelling, or blocking the ureters by sludge-like material.There was a full recovery of kidney function following cessation of acetazolamide intake, the removal of obstruction in all the individuals through IV or oral hydration, and avoidance of concurrent ischemic condition or the need for drugs, such as NSAIDs (28-31).

It is needless to say that occurrence of AKI after cardiac surgery is mostly secondary to a reduction in renal blood flow, leading to ischemic damages (32-34). The previous studies have demonstrated the role of carbonic anhydrase activity in acute recovery following renal ischemia-reperfusion injury (35, 36).

These findings may suggest two roles for carbonic anhydrase; the first one is the major role of carbonic anhydrase in decreasing ischemic injury by influencing renal oxygen homeostasis; the second one is related to natriuretic response to inhibit carbonic anhydrase and activation of tubuloglomerular feedback mechanism, as well as increasing proximal tubular pressure, leading to a decrease in renal blood flow, and glomerular filtration rate. 

Therefore, it may not be reasonable to assert that acetazolamide administration before surgery is a nephroprotective method against ischemic injuries.

The previous studies have shown that acidic pH of tubular fluid influences ROS activity (16, 37, 38), resulting in an increase in the level of inflammatory markers and the exacerbated renal injury (39), as confirmed in the study by Assadi, who found that exposure to acetazolamide 2 h before and 12 h after the contrast agent administration could preferentially scavenge ROS by alkalinizing tubular fluid (16).

Since the effect of acetazolamide on inhibition of bicarbonate tubular reabsorption can be estimated simply by monitoring serum bicarbonate level (40). Coincidently, our results showed that serum bicarbonate level was significantly lower in the acetazolamide group than the control group just on the first day after CABG surgery, which could confirm alkalinization of renal tubular.

However, our study findings showed no significant difference in post-CABG CRP level between the two groups. As it seems to prevent activation of cytokine-induced inflammatory mediators, it may need to maintain alkalinization of tubular fluid status for longer periods.

There were some limitations in this study. Firstly, because of high cost and resource limitations regarding the use of parenteral acetazolamide in our institution, oral forms of the medication were used, which has a different bioavailability than its parenteral form. Second, since critical conditions could influence the absorption and bioavailability of acetazolamide, it is recommended to perform more clinical trials with varying acetazolamide doses to determine the precise dosage of this agent preventing AKI after CABG surgery. Also, it is recommended to conduct similar studies with a larger sample size to generalize the results to the other populations better.

**Table 1:** KDIGO classification

**Stage of ** **acute kidney injury**	**Serum Creatinine criteria**	**Urine output criteria**
1	1.5–1.9 times baseline	< 0.5 mL/kg/h for 6–12 h
	≥0.3 mg/dL (≥26.5 μmol/L) increase within 48 h	
2	2.0–2.9 times baseline	< 0.5 mL/kg/h for ≥12 h
3	≥3.0 times baseline	< 0.3 mL/kg/h for ≥24 h
	Increase in serum creatinine to ≥4.0 mg/dL (≥353.6 μmol/L)	Anuria for ≥12 h
Initiation of renal replacement therapy	

**Table 2:** Baseline characteristics of the patients

**Variables**	**Control (n = 65)**	**Acetazolamide (n = 65)**	***p*** **-value**
Age (year), mean ± SD	61 ± 11	60 ± 10	0.706
Sex (male), n (%)	48(73.84)	54(83.07)	0.201
body mass index (kg/m2), mean ± SD	26 ± 3	26 ± 4	0.625
Drug/food allergy, n (%)	0	2(3.07)	0.154
Hypertension,n (%)	38(58.46)	35(53.84)	0.596
Diabetes, n (%)	27(41.53)	17(26.15)	0.064
Hyperlipidemia, n (%)	20(30.76)	21(32.30)	0.850
Ischemic heart disease, n (%)	26(40)	22(33.84)	0.467
Stroke, n (%)	5(7.69)	2(3.07)	0.244
Chronic lung disease, n (%)	2(3.07)	4(6.15)	0.403
Smoking, n (%)	30(46.15)	30(46.15)	1
Left ventricular ejection fraction %,median(25^th^,75^th^)	50(40, 60)	50(42, 55)	0.962
Beta-receptor blockers, n (%)	25(38.46)	21(32.30)	0.463
Calcium channel blockers, n (%)	8(12.30)	5(7.69)	0.380
ACE inhibitor, ARB, n (%)	22(33.84)	26(40)	0.467
Platelet inhibitors, n (%)	35(53.84)	38(58.46)	0.596
Statin, n (%)	32(49.23)	38(58.46)	0.291
Vasodilator, n (%)	15(23.07)	18(27.69)	0.545
Non-steroidal anti-inflammatory drugs (NSAIDs), n (%)	8(12.30)	16(24.61)	0.07

ACE: Angiotensin-converting enzyme; ARB: angiotensin-receptor blocker.

**Table 3:** Biochemical data before and after CABG surgery in the control and acetazolamide groups

Variables	Control (n = 65)	Acetazolamide (n = 65)	*P*-value
Serum creatinine concentration, before	1.2(1.2-1.3)	1.18(1.17-1.30)	0.341
Serum creatinine concentration, Day1	1.33 ± 0.27	1.43 ± 0.25	0.036
Serum creatinine concentration, Day2	1.33(1.32-1.50)	1.36(1.32-1.52)	0.998
Serum creatinine concentration, Day3	1.22(1.22-1.38)	1.15(1.13-1.43)	0.074
Serum creatinine concentration, Day4	1.18(1.15-1.29)	1.10(1.09-1.37)	0.172
Serum creatinine concentration, Day5	1.18(1.11-1.23)	1.10(1.05-1.30)	0.181
Serum creatinine concentration, Day6	1.16(1.09-1.21)	1.07(1.04-1.28)	0.181
Serum creatinine concentration, Day7	1.12(1.12-1.23)	1.06(1.05-1.28)	0.109
Serum creatinine changes base-day1	-0.09(-0.13, 0.02)	-0.23(-0.26, -0.14)	0.002
Serum creatinine changes base-day2	-0.09(-0.21, -0.05)	-0.11(-0.28, -0.09)	0.615
Serum creatinine changes base-day3	0.00(-0.10, 0.05)	0.09(-0.18, 0.08)	0.074
Serum creatinine changes base-day4	0.08(-0.01, 0.12)	0.12(-0.13, 0.13)	0.247
Serum creatinine changes base-day5	0.07(0.03, 0.17)	0.13(-0.07, 0.17)	0.303
Serum creatinine changes base-day6	0.10(0.05, 0.18)	0.13(-0.04, 0.19)	0.498
Serum creatinine changes base-day7	0.10(0.03, 0.16)	0.15(-0.04, 0.17)	0.208
Blood Ph, Before	7.34(7.30, 7.40)	7.35(7.30, 7.40)	0.772
Blood Ph, 24 h after CABG surgery	7.42(7.40, 7.47)	7.42(7.38, 7.43)	0.027
Absolute change	-0.07 ± 0.08	-0.05 ± 0.10	0.286
Serum bicarbonate level, Before	21.21 ± 2.75	20.94 ± 2.72	0.570
Serum bicarbonate level, 24 h after CABG surgery	25.27 ± 3.37	22.07 ± 3.43	0.00
Absolute change	-4.20(-6.95, -2.5)	-1.30(-3.4, 1.2)	0.00

CABG: coronary artery bypass grafting.

**Table 4:** Post-operative primary and secondary outcomes in 130 studied patients

**Outcome**	**Control (n = 65)**	**Acetazolamide (n = 65)**	***p*** **-value**
Acute kidney injury, n (%)	28(43.07)	29(44.61)	0.860
Need to post-operative RRT	0	0	-
Prolonged ventilation time (hour), median (25^th^,75^th^)	12(8.5,18)	11(8,21)	0.573
Mortality, n (%)	7(10.76)	4(6.15)	0.344
ICU length of stay (day), median (25^th^,75^th^)	6(5,8)	7(5,7)	0.843
Hospital length of stay (day), median (25^th^,75^th^)	9(8,12)	8(7,10)	0.006

Binary characteristics are reported as No (%), and continuous characteristics are reported as median (25th percentile to 75th percentile). RRT: renal

replacement therapy.

**Table 5:** Intra- and post-operative characteristics of the patients in the acetazolamide and control groups

**Characteristic**	**Control (n = 65)**	**Acetazolamide (n = 65)**	***p*** **-value**
Anesthesia duration (hour), mean ± SD	6.40 ± 1.19	6.07 ± 1.33	0.143
Clamp time (minute), mean ± SD	69 ± 28	72 ± 32	0.559
Pump time (minute), mean ± SD	120 ± 39	124 ± 46	0.649
Albumin intake during surgery, n (%)	51(78.4)	50(76.9)	0.833
Albumin intake in ICU, n (%)	43(66.1)	45(69.2)	0.708
Packed cells during surgery, n (%)	42(64.6)	41(63.0)	0.855
Packed cells in ICU, n (%)	29(44.6)	44(67.6)	0.08
Need for inotropes in ICU, n (%)	59(90.7)	61(93.8)	0.510
LVEF after surgery %, median(25^th^,75^th^)	50(40,55)	50(45,55)	0.981
CRP level before surgery(mg/L),median (25^th^,75^th^)	2(2,13)	4(2,13)	0.743
CRP level 24 h after surgery (mg/L),median (25^th^,75^th^)	13(6,24.5)	16(8,26)	0.131
Change in CRP level (mg/L), mean ± SD	8 ± 10	10 ± 12	0.312
Mediastinitis, n (%)	3(4.6)	2(3.0)	0.648
Bleeding requiring surgical reintervention, n (%)	4(6.1)	2(3.0)	0.403
Thrombocytopenia, n (%)	26(40)	29(44.6)	0.594
Fatigue, n (%)	5(7.6)	3(4.6)	0.465
Skin allergy, n (%)	3(4.6)	6(9.2)	0.30

SD: standard deviation; ICU: intensive care unit; LVEF: left ventricular ejection fraction; CRP: C-reactive protein. There was no significant difference

in post-operative LVEF, serum CRP level, adverse effects, and surgery complications between the two groups.

## Conclusion

Our findings revealed that administration of acetazolamide before CABG surgery did not reduce AKI incidence, duration of mechanical ventilation, length of ICU stays, and mortality rate. However, it was found that administration of acetazolamide can reduce the length of hospital stay in the patients undergoing CABG surgery. Although, further high-quality RCTs are needed to clarify the potential renoprotective effect of acetazolamide on AKI incidence after surgery.

## Conflict of interest

The authors declare that they have no conflict of interests

## Author contributions

AG: The first author, collected the cases, and (AG) has analyzed, drafted the manuscript. BS: analyzed the data using SPSS software program. AZ, BM, and KH participated in the design of the study and developed the research question. ZS: drafted the work and revised. DF: coordinated the study, participated in its conception and its design, and reviewed the manuscript. All authors contributed to and have approved the final manuscript.
